# Immunohistochemical expression of TGFβ, E-cadherin and vimentin in benign and malignant neoplasias of canine mammary gland

**DOI:** 10.1186/1753-6561-7-S2-P20

**Published:** 2013-04-04

**Authors:** Erika M Terra, Geórgia M Magalhães, Marcela MP Rodrigues, Renée L Amorim, Noeme S Rocha, Mirela T Costa

**Affiliations:** 1 Department of Clinics and Veterinary Surgery, UNESP, Jaboticabal, SP, Brazil; 2 Department of Veterinary Pathology, UNESP, Jaboticabal, SP, Brazil; 3 Department of Urology, Faculty of Medicine, UNESP, Botucatu, SP, Brazil; 4 Department of Veterinary Pathology, UNESP, Botucatu, SP, Brazil

## Background

Epithelial-mesenchymal transition (EMT) is a fundamental biologic process whereby epithelial cells detach from the surrounding tissue and acquire characteristics of mesenchymal cells, a unique motile, spindle-shaped cell with end-to-end polarity. EMT can be induced or regulated by various growth and differentiation factors; among these, TGFβ has received much attention as a major inducer of EMT during embryogenesis, cancer progression and fibrosis. Our aim was to correlate the immunohistochemical expression of TGFβ, e-cadherin and vimentin in canine mammary tumors.

## Materials and methods

A total of 52 canine mammary tumors, among adenomas (G1, n = 12), non-metastatic carcinomas (G2, n = 24) and metastatic carcinomas (G3, n = 16), were used to evaluate the immunohistochemical expression of TGFβ1, E-cadherin and vimentin. Fisher's Exact Test was used for statistical analysis.

## Results

E-cadherin was not differentially expressed in the three tumor groups. Vimentin expression was significantly higher in malignant neoplasias (G1 *vs.* G2, *p*=*0.019*) and (G1 *vs.* G3 *p*=*0.006*), with no difference in cases with and without metastasis. The expression of TGFβ was significantly higher in adenomas compared to metastatic carcinomas (*p*=*0.01*). There was no difference between adenomas and non-metastatic carcinomas.

## Conclusion

The pathogenesis and the progression of numerous cancers have been attributed, at least in part, to disruption of normal TGFβ signaling. Here, we found that decreased expression of TGFβ in metastatic carcinomas was accompanied by the acquisition of a mesenchymal phenotype, raising the possibility that this cytokine may be involved in EMT in canine mammary neoplasias.

## Financial support

FAPESP.

